# Harnessing Poverty Alleviation to Reduce the Stigma of HIV in Sub-Saharan Africa

**DOI:** 10.1371/journal.pmed.1001557

**Published:** 2013-11-26

**Authors:** Alexander C. Tsai, David R. Bangsberg, Sheri D. Weiser

**Affiliations:** 1 Chester M. Pierce, MD Division of Global Psychiatry, Massachusetts General Hospital, Boston, Massachusetts, United States of America; 2 Harvard Medical School, Boston, Massachusetts, United States of America; 3 Center for Global Health, Massachusetts General Hospital, Boston, Massachusetts, United States of America; 4 Department of Global Health and Population, Harvard School of Public Health, Boston, Massachusetts, United States of America; 5 Mbarara University of Science and Technology, Mbarara, Uganda; 6 Division of HIV/AIDS, San Francisco General Hospital, University of California at San Francisco, San Francisco, California, United States of America

## Abstract

Alexander Tsai and colleagues highlight the complex relationship between poverty and HIV stigma in sub-Saharan Africa, and discuss possible ways to break the cycle.

*Please see later in the article for the Editors' Summary*

**Summary Points**

- Poverty is an important driver of HIV stigma.
- HIV-related morbidity undermines HIV-infected persons' ability to maintain their full economic contributions to family and community life.
- Stigmatization and exclusion from local solidarity networks erode food and livelihood security, which undermine adherence to HIV treatment, further perpetuating this cycle.
- HIV treatment can improve the economic, mental, and social health of HIV-infected persons, but it may not be sufficient to reduce the stigma of HIV.
- Livelihood interventions can reduce the stigma of HIV by directly targeting poverty.

## Introduction

HIV is highly stigmatized throughout sub-Saharan Africa [1],[2]. In studies conducted among general population samples, stigma has been shown to impede uptake of HIV testing and increase sexual risk-taking behavior [3],[4]. Among HIV-infected persons, stigma has also been associated with inhibited serostatus disclosure to sexual partners and potential treatment supporters, delays in HIV antiretroviral therapy (ART) initiation, and ART non-adherence [5],[6],[7]. The stigma of HIV also intensifies the poverty, stress, and insecurity endemic to many resource-limited settings [8], resulting in worsened mental health [9], itself an important determinant of AIDS-related mortality [10]. Until we can better understand how to effectively intervene to reduce the stigma of HIV, it will continue to adversely affect the well-being of HIV-infected persons and undermine both treatment and prevention efforts throughout sub-Saharan Africa.

Given the pressing need for effective intervention, in this Policy Forum article we argue that poverty alleviation should be conceptualized as a potentially powerful yet understudied tool for confronting the stigma of HIV. Beginning with a conceptual discussion of stigma, we draw on qualitative and epidemiological studies to focus our attention on the perceived disability, economic incapacity, and death associated with HIV as key determinants of stigma in numerous sub-Saharan African countries. We then explain how poverty alleviation may help reduce the stigma of HIV. In doing so we borrow from disparate lines of inquiry, including HIV research and programmatic work in sub-Saharan Africa; the medical literature on other stigmatized conditions, such as epilepsy and schizophrenia; and literature from other fields, including anthropology, economics, psychology, and sociology.

## The Stigma of HIV in Sub-Saharan Africa

In many cultural contexts, the stigma of HIV is largely driven by concerns about its symbolic meanings [11]. Gilmore and Somerville [12] argue that stigma is best understood as a social process which involves the exercise of power, along the fault lines of these symbolic meanings, by one group over another. Consistent with their argument, stigma has historically been deployed in a manner that serves to reproduce inequalities that existed even prior to the HIV epidemic, thereby displacing already-marginalized groups even further downward in the status hierarchy (Box 1). For example, during the first decade of widespread awareness about the epidemic in the US, HIV was associated with marginalized groups perceived to be deviant or engaged in deviant behavior, such as injection drug users and men who have sex with men.

**Box 1. The Stigma Process**

- Goffman [70] described stigma as an attribute that is “deeply discrediting” and that, in the eyes of society, reduces someone “from a whole and usual person to a tainted, discounted one” (p. 3).
- Persons with and without the stigmatized attribute are separated into two categories: “them” vs. “us” [71],[72].
- *Internalized stigma* results when stigmatized persons come to accept these inhospitable attitudes as valid, thereby developing self-defacing beliefs and perceptions about themselves [73].
- *Enacted stigma* results when clandestine hostility and/or overt acts of discrimination are directed towards persons specifically because they possess the stigmatized attribute [71].
- In such an environment, even stigmatized persons who are not directly victimized may experience fear in anticipation of being targeted (*felt stigma*) [74].
- These processes are contingent on differentials in power [12],[75].

**Figure 1:**
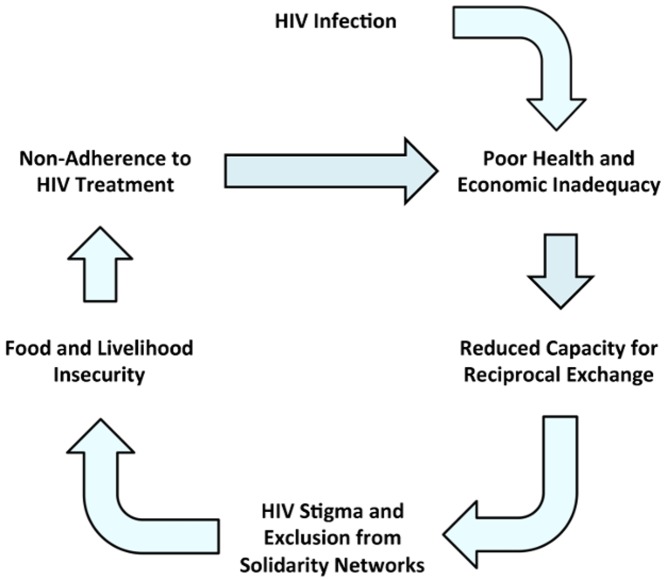
Relationships between HIV, disability, and stigma. HIV infection has adverse health and economic impacts, undermining HIV-infected persons' abilities to maintain their full economic contributions to family and community life and engage in reciprocal exchange. Stigmatization and exclusion from local solidarity networks erodes their food and livelihood security, which in turn undermine their adherence to ART. Non-adherence to ART perpetuates this cycle by further compromising health and economic viability.

Although in some sub-Saharan African countries the stigma of HIV may be partly rooted in its association with perceived deviance, others have argued that such an account insufficiently explains persisting negative attitudes toward HIV-infected persons [13],[14],[15]. We hypothesize that an overlooked—and under-targeted—driver of HIV stigma is its powerful association with disability, economic incapacity, and death. Our hypothesis is grounded in the observation that, in settings of generalized poverty where subsistence agriculture is the norm and where social protection schemes are limited, it is a critical aspect of human sociality to be perceived as sufficiently fit to engage in physical labor [16],[17] and to be perceived as actively contributing to networks of mutual aid [18]. Economic incapacity resulting from HIV-associated morbidity, and the specter of impending premature mortality, call into question HIV-infected persons' abilities to support their households via productive labor or to engage in reciprocal exchange, thereby singling them out for social exclusion (Figure 1).

The mechanism we describe here has been identified as a key culprit explaining the corroded social status of HIV-infected persons in different settings throughout sub-Saharan Africa [19]. Table S1 provides examples of qualitative studies from sub-Saharan Africa in which poverty and inability to engage in reciprocal exchange were cited as drivers of stigma. For example, a young man from a community sample in Zimbabwe portrayed HIV-infected persons as a drain on their communities: “Right now those who are infected are not treated as fellow human beings. They are already declared dead, and regarded as useless as a grave… They mean that these people are no longer able to do anything useful. They say they are just waiting for the day of their death” [20] (p. 2275). These attitudes are embedded in local descriptions of HIV-infected persons who have been labeled “corpses that live” [13] (p. 848). Table S2 lists other dysphemisms that have been applied to HIV-infected persons in countries throughout sub-Saharan Africa.

The conceptual discussion above describes both symbolic and instrumental drivers of stigma [11]. On the one hand, its symbolic component is derived from what HIV symbolizes in specific cultural contexts: disability, economic incapacity, and death. On the other hand, the exclusion of economically inadequate persons from participation in local solidarity networks can be viewed as instrumental, i.e., serving a utilitarian function. This view of stigma emphasizes the instrumental, rather than the symbolic, value of stigma to groups who are in a position to engage in the type of exercise of power described above. The instrumental basis for stigmatization has been elaborated from an evolutionary perspective by social psychologists who treat stigma as a behavioral adaptation designed to solve one of the central problems of human sociality: the need for long-term, cooperative, reciprocal exchange [21],[22]. Persons targeted for stigmatization are simply those who are perceived to violate the norm of reciprocity.

The stigma of HIV can also be understood using concepts borrowed from social capital theory. Persons living in settings of generalized poverty rely heavily on social capital (specifically, bonding and bridging social capital [23]) to access material resources needed for survival. These themes are prominently described in numerous qualitative studies from sub-Saharan Africa [24],[25],[26],[27],[28]. The motivating forces behind social capital are not always altruistic, however, as Portes and Sensenbrenner [29] argued. When social capital is rooted in instrumental rather than altruistic concerns, persons who do not fulfill reciprocity expectations can be excluded. Accordingly, in some communities, fear of the “social death” and exclusion resulting from an HIV diagnosis is more intense than the fear of the disease itself [30].

To date, a number of different approaches to stigma reduction have been attempted. These have largely consisted of educational interventions, aimed at the general population to correct overblown fears about casual transmission risks, or contact interventions in which study participants experience scripted interactions with HIV-infected persons in order to increase tolerance and acceptance [31]. Under the framework described above, these approaches can be thought of as addressing either symbolic or instrumental aspects of HIV stigma. Persons who are exposed to an educational intervention (that demonstrates how the risks of casual transmission are much lower than they previously thought) might be expected to revise their instrumental concerns about HIV. Similarly, persons who are exposed to HIV-infected persons through a contact intervention might be inclined to revise their symbolically grounded views about the humanity of HIV-infected persons (a process termed “recategorization” [32]). These approaches, however, do not necessarily consider the underlying functional aspects of HIV stigma.

## ART Scale-Up as a Necessary but Not Sufficient Stigma Reduction Strategy

If an important driver of HIV stigma is its powerful association with disability, economic incapacity, and death, then ART might receive an alternative reading as an anti-stigma intervention. Although ART is deployed at the level of the *individual*, it should also be regarded as a structural intervention aimed at altering the *context* in which HIV-infected persons interact with their HIV-uninfected peers [33]. We conceptualize HIV treatment as increasing the perceived social value of HIV-infected persons, by detaching the perceived inevitability of death from HIV infection and by directly addressing societal assessments about one's ability to adhere to norms of reciprocity. Consistent with this hypothesis, epidemiologic and econometric studies have demonstrated that the availability of and perceived access to ART have altered general population expectancies about longevity in Malawi [34],[35] and diminished negative attitudes towards HIV-infected persons in Botswana [2]. In fact, an early argument advanced in support of ART scale-up was that doing so would reduce stigma and enhance prevention efforts [36].

For HIV-infected persons, the past decade of ART expansions has vindicated these claims. Program implementers at the non-governmental organization Partners in Health, for example, reported that after the introduction of ART in Haiti, they observed greater demand for HIV testing and counseling, and their patients reported fewer HIV-related discriminatory events and increased social integration [36],[37]. As shown in Table S1, in qualitative studies conducted in countries throughout sub-Saharan Africa, HIV-infected persons initiating ART have similarly described a constellation of benefits, including improved economic productivity, greater self-efficacy, and reduced internalized stigma [38]. These findings have been confirmed in larger-scale epidemiological studies as well [9],[39],[40],[41],[42],[43],[44].

Yet, while ART has undeniably improved the economic, mental, and social health of HIV-infected persons, two factors may hinder its ability to level stigmatizing beliefs in the general population. First, accurate knowledge about ART efficacy may not evenly follow the contours of treatment expansion. Over the past decade, with substantial investments in educational HIV-related messages promoted through radio, television, and/or print media, overall levels of accurate HIV-specific knowledge have improved slightly but remain low in general population surveys conducted throughout sub-Saharan Africa [45]. Similarly, local beliefs about ART availability or ART efficacy may also lag, thereby perpetuating the assumed association between HIV and disability, economic incapacity, and death. This could potentially explain why uptake of HIV testing throughout sub-Saharan Africa, while increasing over time, has remained persistently low despite large-scale expansions in access to ART [46]. Refusal of treatment despite eligibility has also been described [47].

Second, ART alone may not be sufficient to decrease the stigma of HIV. Despite expansions in access to ART at no charge, many patients present for treatment initiation late in the disease stage. As a result, even when they experience dramatic recovery of physical function, they may return to work at a lower intensity compared to their pre-disease state [17],[18],[48],[49]. In addition, even HIV-infected persons who are able to resume working at full capacity have nonetheless voiced difficulties in rebounding to the same overall level of economic well-being. While trying to cope with HIV-associated morbidity before treatment initiation, they may have had to adopt coping strategies such as selling off productive assets or shifting from high-return to low-return activities, while ultimately undermining their own financial viability in the long term. These themes are prominent in qualitative studies [16],[17],[19],[27],[49] and have been confirmed in larger-scale epidemiological studies [50].

## “ART-Plus”: Harnessing Poverty Alleviation to Reduce the Stigma of HIV

Considering the important limitations in our current portfolio of anti-stigma interventions [31], we believe poverty alleviation—and, specifically, livelihood interventions—should also be considered in conjunction with ART to help further reduce the stigma of HIV. We recognize that the idea of “ART-plus” does not, at present, have widespread adherents. For example, a recently published Cochrane Collaboration protocol for a planned systematic review of interventions to reduce HIV stigma lists an exhaustive catalogue of educational anti-stigma interventions, including “lectures, group discussions, individual education, radio, television, print (newspapers, magazines, booklets, leaflets, posters, pamphlets), films, documentaries, billboards, folk media (such as street dramas), or a combination of these” [51] (p. 4) but does not provide any indication that livelihood interventions will be examined.

The hypotheses we describe in this article are consistent with an argument advanced by Castro and Farmer [37], who prominently called attention to the ways in which the stigma of HIV is mainly determined by large-scale social forces such as poverty and racial and gender inequality. Drawing on their programmatic and research experience with HIV-infected persons in Haiti, they write, “poverty, already representing an almost universal stigma, will be the primary reason that poor people living with HIV suffer from greater AIDS-related stigma” (p. 55). Conceptually, this line of argument suggests that HIV-related stigmatization may at least be partially conditioned on poverty, extending the cognitive and psychological models described above to emphasize how power relations and economic and other structural factors influence community responses to HIV. Qualitative studies from Brazil [52], Zambia [53], and Zimbabwe [54] have generated similar conclusions.

Non-governmental organizations have already moved forward with programs to address these issues [41],[55],[56],[57],[58]. Indeed, the summary term we have adopted here, “ART-plus,” is borrowed from a Peruvian non-governmental organization's pilot program that employed a comprehensive HIV treatment strategy with both ART and socioeconomic support [55]. Yet although the multiple lines of inquiry described in this article provide sufficient groundwork to justify the implementation of experimental or quasi-experimental studies, to date the available evidence in this area has been exclusively observational. In qualitative studies of HIV-infected persons participating in integrated livelihood intervention programs in different sub-Saharan African settings, study participants reported less stigma, increased social integration, and improved status in the community (Table S1). These changes were explicitly linked to their improved economic standing, as one program staff member observed: “Some participants…have gained [a] reputation within their communities…Because of the cow, because of the income they are getting, they have been taking on positions of responsibility in their communities, in churches, in schools, on boards…they are now getting less stigmatized in a sense… [the community] can see them managing their own issues, managing themselves, surviving…” [58] (p.e26117). In other words, by availing themselves of economic opportunities brought about through these interventions, HIV-infected persons were able to construct “positive identities” for themselves in much the same fashion as when they were first restored to health after initiating ART [54] (p. 1008).

Additional evidence comes from a longitudinal study with matched controls, in which Muñoz and colleagues [59] studied the psychosocial impacts of a comprehensive social support intervention among 120 HIV-infected persons receiving ART in Peru. At 24-month follow-up, participants in the intervention group reported less stigma and perceived greater social support. Because only ten of these actually received individually tailored microfinance assistance, there was limited ability to draw inferences about the incremental stigma-reducing effects of the microfinance assistance component of the intervention. However, studies of similar types of interventions based on participant samples unselected for HIV seropositivity have shown statistically significant impacts on mental health [60],[61] and social integration [62], thereby lending further plausibility to the effects shown by Muñoz and colleagues [59].

**Figure 2:**
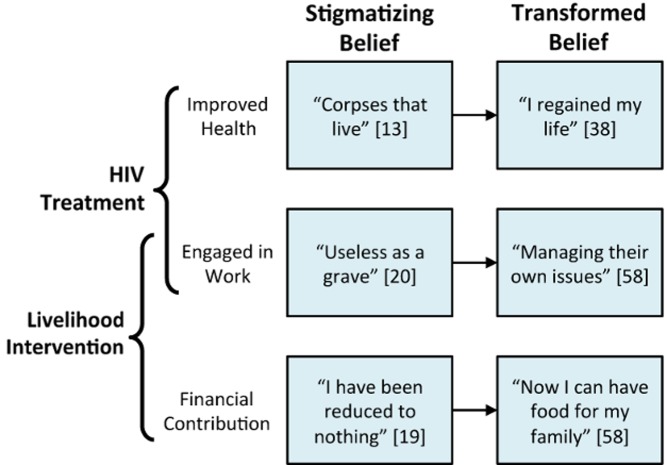
Transforming HIV-stigmatizing beliefs through ART-plus. ART manages the disability incurred through HIV infection, undermining the view of HIV-infected persons as economically inadequate members of the community and permitting them to re-engage in productive activity. Livelihood interventions, such as combined financial literacy and microenterprise management programs, may additionally create new modes of engaging in productive activity, such as new trades or new enterprise development. These financial contributions are welcomed within local solidarity networks, and the norm of reciprocity is upheld.

A simplified diagram summarizing our hypotheses about how ART-plus revises HIV-stigmatizing beliefs is shown in Figure 2. In our account, ART-plus addresses internalized stigma, by enhancing the HIV-infected person's self-efficacy and self-esteem. This aspect of our framework is most closely related to the conceptual model and empirical study by Rosenfield [63], who identified links between economic rehabilitation, self-efficacy, social status, and subjective quality of life among functionally impaired persons with mental illness in the US. Further, if the objective is “recategorization” (as described above in the context of contact interventions), ART-plus also offers a different type of recategorization to address enacted stigma, by encouraging a view of HIV-infected persons not as a net drain on their communities but rather as productive members of society who are “managing their own issues” [58] (p.e26117).

## Limitations of the ART-Plus Approach

We recognize several potential pitfalls with this proposed ART-plus approach to stigma reduction. First, while we believe that the perceived association between HIV and disability, economic incapacity, and death is a primary driver of stigma, we also recognize that stigma is complex and may be driven by many other factors depending on the cultural context. Despite dramatic expansions in access to ART in urban South Africa, for example, a longitudinal study by Maughan-Brown [64] showed continued increases in symbolic stigma centered around the association of HIV with promiscuity. Similarly, a common prominent theme in several qualitative studies from Tanzania was a negative perception of HIV-infected persons that persisted specifically *because of* ART's effects on prolonging life [65],[66]. Study participants voiced concerns that ART changed HIV-infected persons from diseased but visibly ill and incapable of sexual risk-taking behavior, to diseased but healthy-appearing potential vectors of sexual transmission. In settings where such symbolism is a secondary but nonetheless substantial driver of stigma, even if a combined ART-plus intervention could undo the association between HIV, disability, and economic incapacity, it would still “leave the spectre of illicit sex untouched” [67] (p. 643).

A second issue that poses a dilemma for the implementation of our proposed strategy, without undermining the strategy itself, is that precisely targeting this type of intervention may be politically difficult. Making economic resources available specifically to HIV-infected persons in the context of widespread poverty may generate resentment not unlike the antipathy directed towards welfare recipients in the US beginning in the 1970s and continuing to the present day. For example, participants in a qualitative study from South Africa intimated that welfare fraud was widespread among HIV-infected persons receiving disability grants [68]. Because of these reasons, there is a consensus that broad-based targeting schemes (e.g., based on poverty and multiple vulnerability criteria) for cash transfer interventions should be used rather than procedures focused on HIV-affected status or orphanhood alone [69]. However, interventions that focus on livelihoods, such as animal husbandry training or microfinance assistance for small enterprises, may be perceived in the community to be more sustainable than interventions that provide direct food or cash transfers and therefore engender less resentment [58].

## Conclusion

Taken together, these related strands of inquiry build a strong case for considering livelihood interventions as a compelling stigma reduction strategy with the potential to improve the well-being of HIV-infected persons throughout sub-Saharan Africa. Although our framework has not been rigorously tested in an experimental context, we believe the corroborating evidence from anthropology, economics, psychology, and sociology is strongly suggestive. It would be imprudent for us to suggest that prevailing approaches to stigma should be abandoned despite their uneven effectiveness to date. Future studies may find that the most effective approaches to stigma reduction adopt synergistic strategies that target both the stigmatized (through livelihood interventions) as well as the stigmatizers (through educational or contact interventions).

## Supporting Information

Table S1 Poverty and lack of reciprocal exchange as important drivers of stigma, described in qualitative studies conducted in sub-Saharan Africa.
(DOCX)

Table S2 Local dysphemisms for HIV-infected persons.
(DOCX)
