# Supplementary material for: Harnessing Poverty Alleviation to Reduce the Stigma of HIV in Sub-Saharan Africa
Source: PLoS Med. 2013 Nov 26;10(11):e1001557. doi: 10.1371/journal.pmed.1001557 (PMC3841100; doi:10.1371/journal.pmed.1001557)
Supplement: Table S1 — Poverty and lack of reciprocal exchange as important drivers of stigma, described in qualitative studies conducted in sub-Saharan Africa. (DOCX) [file pmed.1001557.s001.docx]

| **Table S1.** Poverty and lack of reciprocal exchange as important drivers of stigma, described in qualitative studies conducted in sub-Saharan Africa | |
| --- | --- |
|  | |
| **Countries** | **Themes** |
| Burkina Faso [w1], Ghana [w2], Namibia [w3], Nigeria [w4], Uganda [w5] | Loss of status resulting from morbidity-related inability to contribute to reciprocal social networks or fulfill other social obligations |
| Tanzania [w6], Uganda [w7,w8], Zambia [w9], Zimbabwe [w6] | Poverty as a key driver of stigmatizing attitudes against HIV-infected persons |
| Botswana [w10], Kenya [w11], Malawi [w12], Nigeria [w13,w14], Tanzania [w13,w15], Uganda [w13,w16,w17, w18,w19], Zimbabwe [w20] | HIV treatment preserving the physical capacity for contributing to reciprocal social networks or fulfilling other social obligations |
| South Africa [w6,w21,w22], Tanzania [w6], Uganda [w23], and Zimbabwe [w6] | Appearance of healthiness while on treatment as a key factor in sustaining social relationships |
| South Africa [w24,w25], Tanzania [w26], Uganda [w27] | Improved self-image and hope related to improved health while on treatment |
| Kenya [w28,w29], Uganda [w30,w31] | Income generating activities resulting in self-sufficiency, improved self-esteem, and increased standing within the community |

WEB REFERENCES

w1. Bila B, Kouanda S, Desclaux A (2008) Des difficultés économiques à la souffrance sociale des personnes vivant avec le VIH au Burkina Faso. Cahiers Sante 18: 187-191.

w2. Kwansa BK (2013) Safety in the midst of stigma: experiencing HIV/AIDS in two Ghanaian communities. Leiden: African Studies Centre.

w3. Thomas F (2006) Stigma, fatigue and social breakdown: exploring the impacts of HIV/AIDS on patient and carer well-being in the Caprivi Region, Namibia. Soc Sci Med 63: 3174-3187.

w4. Hilhorst T, van Liere MJ, Ode AV, de Koning K (2006) Impact of AIDS on rural livelihoods in Benue State, Nigeria. SAHARA J 3: 382-393.

w5. Kaler A, Alibhai A, Kipp W, Rubaale T, Konde-Lule J (2012) Walking corpses and kindly neighbors: retrospective accounts of AIDS stigma in western Uganda. World J AIDS 2: 174-182.

w6. Maman S, Abler L, Parker L, Lane T, Chirowodza A, et al. (2009) A comparison of HIV stigma and discrimination in five international sites: the influence of care and treatment resources in high prevalence settings. Soc Sci Med 68: 2271-2278.

w7. Monico SM, Tanga EO, Nuwagaba A, Aggleton P, Tyrer P (2001) Uganda: HIV and AIDS-related discrimination, stigmatization and denial. Geneva: Joint United Nations Programme on HIV/AIDS.

w8. Wyrod R (2011) Masculinity and the persistence of AIDS stigma. Cult Health Sex 13: 443-456.

w9. Bond V (2006) Stigma when there is no other option: understanding how poverty fuels discrimination toward people living with HIV in Zambia. In: Gillespie S, editor. AIDS, poverty, and hunger: challenges and responses. Washington, D.C.: International Food Policy Research Institute. pp. 181-197.

w10. Nam SL, Fielding K, Avalos A, Dickinson D, Gaolathe T, et al. (2008) The relationship of acceptance or denial of HIV-status to antiretroviral adherence among adult HIV patients in urban Botswana. Soc Sci Med 67: 301-310.

w11. Izugbara CO, Wekesa E (2011) Beliefs and practices about antiretroviral medication: a study of poor urban Kenyans living with HIV/AIDS. Sociol Health Illn 33: 869-883.

w12. Johnson J (2012) Life with HIV: ‘stigma’ and hope in Malawi’s era of ARVs. Africa (Lond) 82: 632-653.

w13. Ware NC, Idoko J, Kaaya S, Biraro IA, Wyatt MA, et al. (2009) Explaining adherence success in sub-Saharan Africa: an ethnographic study. PLoS Med 6: e11.

w14. Okoror TA, Falade CO, Olorunlana A, Walker EM, Okareh OT (2013) Exploring the cultural context of HIV stigma on antiretroviral therapy adherence among people living with HIV/AIDS in southwest Nigeria. AIDS Patient Care STDs 27: 55-64.

w15. Watt MH, Maman S, Earp JA, Eng E, Setel PW, et al. (2009) “It’s all the time in my mind”: facilitators of adherence to antiretroviral therapy in a Tanzanian setting. Soc Sci Med 68: 1793-1800.

w16. Wagner G, Ryan G, Huynh A, Kityo C, Mugyenyi P (2009) A qualitative analysis of the economic impact of HIV and antiretroviral therapy on individuals and households in Uganda. AIDS Patient Care STDS 23: 793-798.

w17. Nyanzi-Wakholi B, Lara AM, Watera C, Munderi P, Gilks C, et al. (2009) The role of HIV testing, counselling, and treatment in coping with HIV/AIDS in Uganda: a qualitative analysis. AIDS Care 21: 903-908.

w18. Russell S, Seeley J (2010) The transition to living with HIV as a chronic condition in rural Uganda: working to create order and control when on antiretroviral therapy. Soc Sci Med 70: 375-382.

w19. Nyanzi-Wakholi B, Lara AM, Munderi P, Gilks C (2012) The charms and challenges of antiretroviral therapy in Uganda: the DART experience. AIDS Care 24: 137-142.

w20. Campbell C, Skovdal M, Madanhire C, Mugurungi O, Gregson S, et al. (2011) “We, the AIDS people...”: how antiretroviral therapy enables Zimbabweans living with HIV/AIDS to cope with stigma. Am J Public Health 101: 1004-1010.

w21. Mfecane S (2012) Narratives of HIV disclosure and masculinity in a South African village. Cult Health Sex 14 Suppl 1: S109-121.

w22. Gilbert L, Walker L (2009) “They (ARVs) are my life, without them I’m nothing”--experiences of patients attending a HIV/AIDS clinic in Johannesburg, South Africa. Health Place 15: 1123-1129.

w23. Mbonye M, Nakamanya S, Birungi J, King R, Seeley J, et al. (2013) Stigma trajectories among people living with HIV (PLHIV) embarking on a life time journey with antiretroviral drugs in Jinja, Uganda. BMC Public Health 13: 804.

w24. Zuch M, Lurie M (2012) ‘A virus and nothing else’: the effect of ART on HIV-related stigma in rural South Africa. AIDS Behav 16: 564-570.

w25. Fried J, Harris B, Eyles J (2012) Hopes interrupted: accessing and experiences of antiretroviral therapy in South Africa. Sex Transm Infect 88: 147-151.

w26. Roura M, Urassa M, Busza J, Mbata D, Wringe A, et al. (2009) Scaling up stigma? The effects of antiretroviral roll-out on stigma and HIV testing. Early evidence from rural Tanzania. Sex Transm Infect 85: 308-312.

w27. Seeley J, Russell S (2010) Social rebirth and social transformation? Rebuilding social lives after ART in rural Uganda. AIDS Care 22: 44-50.

w28. Datta D, Njuguna J (2008) Microcredit for people affected by HIV and AIDS: insights from Kenya. SAHARA J 5: 94-102.

w29. Pandit JA, Sirotin N, Tittle R, Onjolo E, Bukusi EA, et al. (2010) Shamba Maisha: a pilot study assessing impacts of a micro-irrigation intervention on the health and economic wellbeing of HIV patients. BMC Pub Health 10: 245.

w30. Yager JE, Kadiyala S, Weiser SD (2011) HIV/AIDS, food supplementation and livelihood programs in Uganda: a way forward? PLoS One 6: e26117.

w31. Wagner G, Rana Y, Linnemayr S, Balya J, Buzaalirwa L (2012) A qualitative exploration of the economic and social effects of microcredit among people living with HIV/AIDS in Uganda. AIDS Res Treat: Art. #318957, 7 pages. doi: 10.1155/2012/318957.
