# Supplementary material for: Harnessing Poverty Alleviation to Reduce the Stigma of HIV in Sub-Saharan Africa
Source: PLoS Med. 2013 Nov 26;10(11):e1001557. doi: 10.1371/journal.pmed.1001557 (PMC3841100; doi:10.1371/journal.pmed.1001557)
Supplement: Table S2 — Local dysphemisms for HIV-infected persons. (DOCX) [file pmed.1001557.s002.docx]

| **Table S2.** Local dysphemisms for HIV-infected persons | |
| --- | --- |
|  | |
| **Countries** | **Local descriptions** |
| Ghana | “sickness of the dead but alive” [w1] (p.78) |
| Malawi | “you were finished long ago” / “you’re already dead” [w2] (p.284) |
| South Africa | “like a ghost: dead while alive” [w3] (p.228) |
|  | “this thing is already dead” [w4] (p.S116) |
|  | “corpses that live” [w5] (p.848) |
|  | “a waiting room for death” [w6] (p.25) |
| Uganda | “someone who is going to die any time” [w7] (p.1749) |
| South Africa, Tanzania, Zimbabwe | “already corpses” [w8] (p.2275) |

WEB REFERENCES

w1. Dapaah JM (2012) HIV/AIDS treatment in two Ghanaian hospitals: experiences of patients, nurses, and doctors. Leiden: African Studies Centre.

w2. Peters PE, Kambewa D, Walker PA (2010) Contestations over “tradition” and “culture” in a time of AIDS. Med Anthropol 29: 278-302.

w3. Wood K, Lambert H (2008) Coded talk, scripted omissions: the micropolitics of AIDS talk in an affected community in South Africa. Med Anthropol Q 22: 213-233.

w4. Mfecane S (2012) Narratives of HIV disclosure and masculinity in a South African village. Cult Health Sex 14 Suppl 1: S109-121.

w5. Niehaus I (2007) Death before dying: understanding AIDS stigma in the South Africa lowveld. J S Afr Stud 33: 845-860.

w6. Zuberi F (2005) “If you (be)come HIV positive, you will lose your human rights.” HIV/AIDS stigma and human rights: a localised investigation of Hammanskraal communities. A report of the Tswelopele Research Project of the Centre for the Study of AIDS. In: Viljoen F, editor. Righting stigma: exploring a rights-based approach to addressing stigma. Pretoria: University of Pretoria. pp. 12-49.

w7. Medley AM, Kennedy CE, Lunyolo S, Sweat MD (2009) Disclosure outcomes, coping strategies, and life changes among women living with HIV in Uganda. Qual Health Res 19: 1744-1754.

w8. Maman S, Abler L, Parker L, Lane T, Chirowodza A, et al. (2009) A comparison of HIV stigma and discrimination in five international sites: the influence of care and treatment resources in high prevalence settings. Soc Sci Med 68: 2271-2278.
